# Protective effect of celastrol on type 2 diabetes mellitus with nonalcoholic fatty liver disease in mice

**DOI:** 10.1002/fsn3.1917

**Published:** 2020-10-12

**Authors:** JuanJuan Sun, Hui‐juan Wang, Jun Yu, TingTing Li, YiDi Han

**Affiliations:** ^1^ The Second District of Hepatopathy Qingdao No. 6 People's Hospital Qingdao China

**Keywords:** celastrol, cell apoptosis, fatty liver disease, nonalcoholic, Nrf2/HO‐1 pathway, type 2 diabetes mellitus

## Abstract

To investigate the protective effects of celastrol on mice with type 2 diabetes mellitus (T2DM) and nonalcoholic fatty liver disease (NAFLD), and to explore its underlying mechanism. The levels of low‐density lipoprotein cholesterol (LDL‐C), high‐density lipoprotein cholesterol (HDL‐C), total cholesterol (TC), and triglyceride (TG) in serum were tested. Malondialdehyde (MDA) and superoxide dismutase (SOD), GOT, and GPT in serum were also detected. The histopathological changes of liver tissues were observed by HE staining. The apoptosis cell number of liver tissues was measured by TUNEL staining. Nrf‐2 and HO‐1 protein and mRNA expression were evaluated by IHC, WB, and RT‐PCR assay. Celastrol had effects to depress TG, TC, LDL‐C, GPT, GOT, and MDA concentration and increase HDL‐C and SOD concentration (*p *< .05, respectively) with dose‐dependent. Compared with model group, apoptosis cell number was significantly depressed in Cel‐treated groups with dose‐dependent (*p *< .05, respectively). Nrf‐2 and HO‐1 mRNA and protein expressions were significantly improved in Cel‐treated groups with dose‐dependent (*p *< .05, respectively). Celastrol can inhibit the oxidative stress reaction and liver cell apoptosis via regulation Nrf2/HO‐1 pathway in T2DM mice with NAFLD.

## Graphical abstract

In this work, we clearly discuss celastrol' effects in T2DM mice with NAFLD treatment.

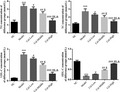

## 1 INTRODUCTION

Type 2 diabetes mellitus (T2DM) is a metabolic disease closely related to obesity. Elevated levels of blood glucose lead to serious damage to the heart, brain, blood vessels, nervous system, and digestive system, and even threaten the life of the patients over time (Byrne & Targher, 2015; Canfora et al., 2019; Tilg et al., 2017). Clinical studies have found that more than 50% of patients with T2DM are complicated with nonalcoholic fatty liver disease (NAFLD), which enhances the development of insulin resistance and impairs the liver function, further aggravating the condition (Ma et al., 2018; Scorletti & Byrne, 2016).

Celastrol (Cel) is a monomer isolated from plants of the genus Tripterygium (a traditional Chinese medicine) and Pteridium. Cel is a pentacyclic triterpenoid pigment with potent pharmacological activities including anti‐inflammation, immunosuppression, inhibition of angiogenesis, and antitumor (Zhan et al., 2018). Studies have shown that Cel can inhibit the production of inflammatory factors, such as IL‐1, TNF‐α, IL‐6, and IL‐8, and block the biological activities of heat shock protein 90 (Hsp90) by interacting with chaperones Cdc37 and p23 (Chadli et al., 2010), thereby protecting cardiomyocytes against injury (Aceros et al., 2019). However, whether Cel plays a role in T2DM with NAFLD remains unclear.

In the present study, the protective effect of Cel on T2DM with NAFLD in mice and its underlying mechanism was investigated, with a view to finding more natural agents for the treatment of T2DM with NAFLD.

## 2 MATERIALS AND METHODS

### 2.1 Experimental animals

Specific pathogen‐free (SPF) C57BL/6 male mice used in this study, 8 weeks old, weighing 20–25 g, were purchased from Beijing Vital River Laboratory Animal Technology Co. Ltd. (Production license No.: SCXF (Beijing) 2016‐0011) and housed in the Animal Experimental Center of Shandong Medical University. All mice were fed normally for 1 week before the experiment for adaption in an animal room at 20–25°C with a relative humidity of 50% ± 5% and a 12:12 hr light and dark cycle. All the mice were allowed free access to food and water.

### 2.2 Reagents

Cel was purchased from Shanghai Yuanye Company (purity >97%); high‐fat feed was provided by Chengdu Dossy Experimental Animal Co. Ltd.; detection kits for total cholesterol (TC), triacylglycerol (TG), high‐density lipoprotein cholesterol ( HDL‐C) and low‐density lipoprotein cholesterol (LDL‐C) were provided by Shanghai Mind Biological Engineering Co., Ltd.; detection kits for aspartate aminotransferase (GOT), alanine aminotransferase (GPT), superoxide dismutase (SOD), malondialdehyde (MDA) were provided by Nanjing Jiancheng Bioengineering Research Institute; terminal in situ nick end labeling (TUNEL) kit was provided by Boehringer Mannheim; and anti‐Nrf‐2, OH‐1 and GAPDH antibodies were purchased from Abcam.

### 2.3 Instruments

AU480 automatic biochemical analyzer (Beckman) and LSM 540 SYSTEM laser confocal microscope (Zeiss) were used in this study.

## 3 METHODS

### 3.1 Animal modeling and grouping

Animal model of T2DM with NAFLD was established by streptozotocin and high‐fat diet as previously described (Tripathi et al., 2019; Wang et al., 2019). Briefly, of 45 C57BL/6 mice, 9 were randomly selected as the normal control group (NC) and fed with the normal diet; and the remaining 36 mice were fed with high‐fat diet for 4 weeks, followed by intraperitoneal injection of streptozotocin solution (30 mg/kg, dissolved in citrate solution). Animals in the NC group were injected with equal volume of citrate solution. Three days later, fasting blood glucose in venous blood was measured in mice fed with high‐fat diet, and the model of T2DM was successfully established if the fasting blood glucose was higher than 11.1 mmol/L for three consecutive days. Mice were fed with high‐fat diet till the end of the 8th week and the model mice of T2DM combined with NAFLD were obtained. The established model mice were randomly divided into the model control (Model) group, the high‐dose Cel group, the medium‐dose Cel group, and low‐dose Cel group (*N* = 9 each group). Animals in the Cel groups were given Cel at 10, 50, and 100 mg/kg respectively, by gavage and those in the Model group and the NC group were given the same volume of distilled water by gavage daily for 6 weeks. Then fasting serum and the liver tissue were collected for analysis.

### 3.2 Determination of serum lipid metabolism and liver function measurements

After fasting for 12 hr, blood samples were collected from the eyeballs and the serum was collected after centrifugation at 3,000 *g*/min for 10 min. Serum TC, TG, HDL‐C, and LDL‐C (lipid metabolism) and serum GOT and GPT (liver function) were determined by automatic biochemical analyzer according to the instructions provided with the respective detection kit.

### 3.3 Detection of serum oxidative stress indexes

After fasting for 12 hr, blood samples were collected from the eyeballs and the serum was collected after centrifugation at 3,000 *g*/min for 10 min. The SOD was detected by the xanthine oxidation method and MDA was detected by the thiobarbituric acid method strictly according to the instructions provided with the respective detection kit.

### 3.4 Changes of liver pathological morphology and liver index in mice by hematoxylin eosin (HE) staining

After blood sampling, the animals in each group were weighed and then sacrificed to collect liver tissues. Paraffin tissue sections were routinely prepared from the liver tissue and HE staining was performed to observe the pathological morphological changes in these liver tissues.

### 3.5 Hepatocyte apoptosis assay by TUNEL

Liver sections were used to conduct routine TUNEL staining. Nuclei in the normal liver cells were stained blue and those in apoptotic liver cells were stained brown. Staining was proceeded strictly according to the instructions. Five fields were randomly selected from each section, and cells were counted under an optical microscope. The number of apoptotic cells in each group was calculated.

### 3.6 Immunohistochemistry (IHC)

Liver tissue section on the slide was dewaxed, dehydrated, and incubated with 3% H_2_O_2_ at room temperature (RT) in dark for 25 min. After being washed 3 times for 5 min each time with PBS, the slide was blocked with 3% BSA for 30 min at RT, followed by incubation with primary antibody overnight at 4°C. The slide was washed 3 times with PBS and then incubated with biotin‐labeled secondary antibody at 37°C for 50 min. After being washed 3 times with PBS, the slide was incubated with horseradish peroxidase at 37°C for 30 min and washed 3 times with PBS. DAB solution was added for color reaction. After thoroughly rinsed with running tap water, the slide was counterstained with hematoxylin for 3 min, and then rinsed with running tap water, dehydrated, cleared, and mounted with neutral gum. Finally, the slide was observed under a microscope and image was acquired and analyzed.

### 3.7 Western blot

Liver tissue was lysed and the lysate was denatured and separated by electrophoresis, followed by transfer of protein to the membrane. The membraned was blocked and incubated with primary antibody at 4°C overnight. The membrane was washed with TBST for 3 times and incubated with goat anti‐rabbit HRP antibody at RT for 1 hr. After washed with TBST, ECL detection was conducted and Image J software was used for image analysis.

### 3.8 Detection of gene expression by RT‐PCR

About 100 mg liver tissue frozen at −80°C was taken, and total RNAs were extracted using the Trizol reagent. The quality of RNAs was determined by ultraviolet absorption method, and reversely transcripted into cDNA. cDNA was amplified using the SYBR green method, with 3 duplicates for each sample. All procedures were done on ice. Primer sequences of Nrf‐2 and OH‐1 were designed using the Primer Premier 5.0 software and synthesized by Servicebio Biotechnology Co., Ltd. The housekeeping gene β‐actin was used as an internal reference. The up and downstream primer sequences used were: β‐actin: 5′‐CTATCCTTCTTCGCAT‐3′ and 5′‐ TAATTGTCGCAGATCG‐3′; nrf‐2: 5′‐TCCAGTCAGAAACCAGTGGAT‐3′ and 5′‐GAATGTCTGCGCCAAAAGCTG‐3′; and HO‐1: 5′‐AAGACTGCGTTCCTGCTCAAC‐3′ and 5′‐AAGACTGCGTTCCTGCTCAAC‐3′. Amplification conditions included predenaturation at 95°C for 30 s, 40 cycles of denaturation at 95°C for 5 s and annealing at 65°C for 30 s. At the end of the reaction, the PCR amplification specificity was determined by the dissolution curve analysis. The CT value was read and the relative mRNA expression of the genes of interest was calculated by 2^−ΔΔCT^ method.

### 3.9 Statistical analysis

SPSS 22.0 statistical software was used to analyze the data and Graphpad Prim 7.0 was used to plot the graphs. The measurement data with normal distribution were presented as mean ± *SD*. For paired comparison among groups, one‐way ANOVA with LSD was used when the variance was equal and with Dunnett's *t* test when the variance was unequal. Data with nonnormal distribution were presented as median and interquartile range, and Kruskal Wallis test was used for comparison. A *p* value <.05 indicated statistically significant differences.

## 4 RESULTS

### 4.1 Comparison of general conditions of mice in each group

Mice in the NC group were active, with normal food consumption and drinking, and their fur was clean and glossy; animals in the Model group was significantly less active, with increased food consumption and drinking as well as urine output, and their fur was messy and dull; and the general condition of mice in the Cel‐High, Cel‐Medium, and Cel‐Low groups were improved to different degrees compared with those in the Model group.

### 4.2 Histopathological changes of the liver

**FIGURE 1:**
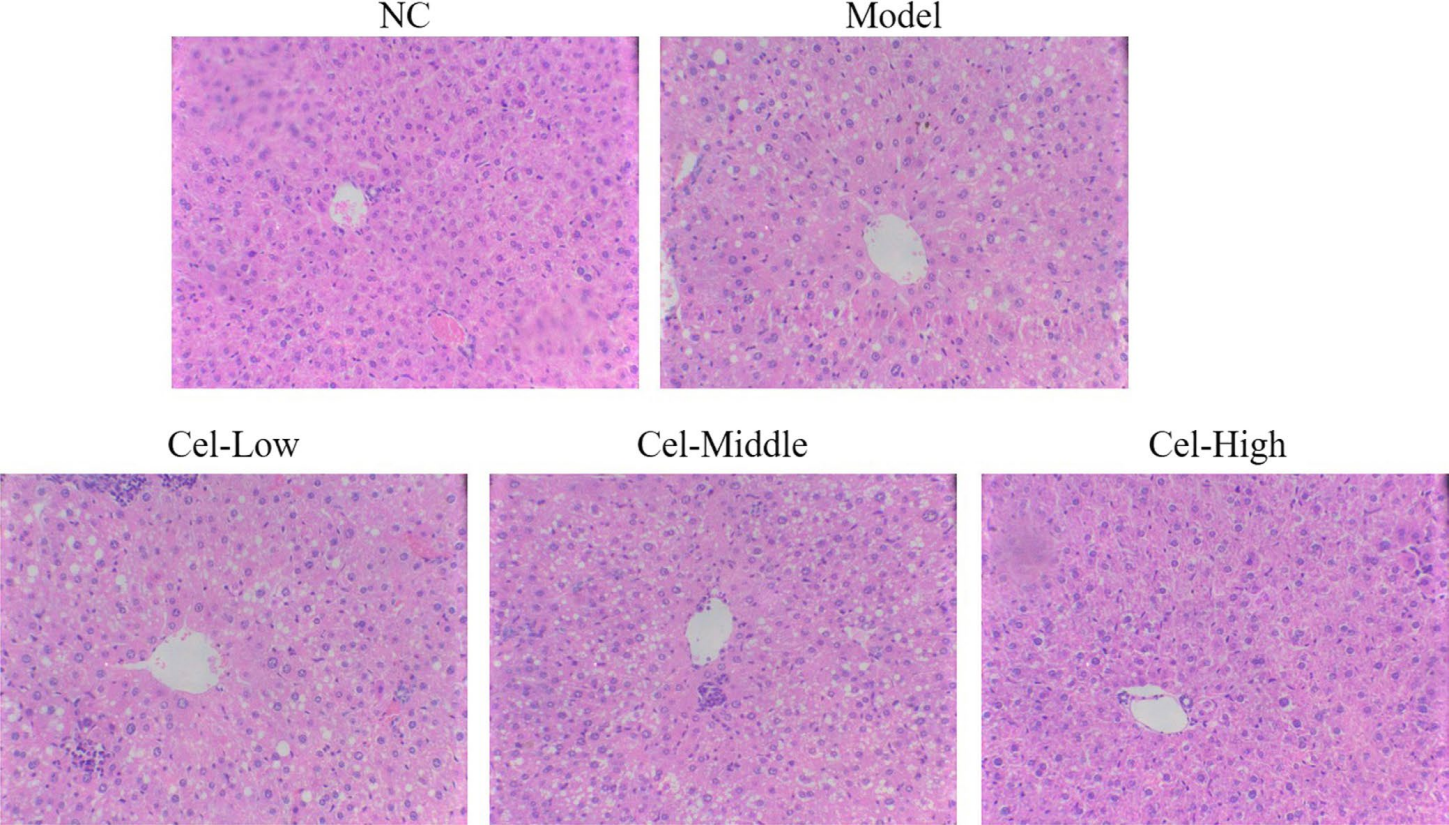
Histopathological charge of difference groups by HE staining (200×). NC: the mice were treated with normal; Model: T2DM mice with NAFLD mice; Cel‐Low: Model mice were treated with 10 mg/kg Cel; Cel‐Middle: Model mice were treated with 50 mg/kg Cel; Cel‐High: Model mice were treated with 100 mg/kg Cel

HE‐stained mouse liver sections were microscopically observed and it was found that the liver lobule structure was normal in the NC group without obvious hepatocyte steatosis and inflammatory cell infiltration; in the animals of the Model group, diffuse hepatocyte steatosis and a large amount of inflammatory cell infiltration were observed in the liver tissue; and compared with the Model group, hepatocyte steatosis and inflammatory cell infiltration in each Cel intervention group were reduced to certain degrees, with the most significant improvement in the Cel‐High group (Figure 1).

### 4.3 Comparison of lipid metabolism indexes

**FIGURE 2:**
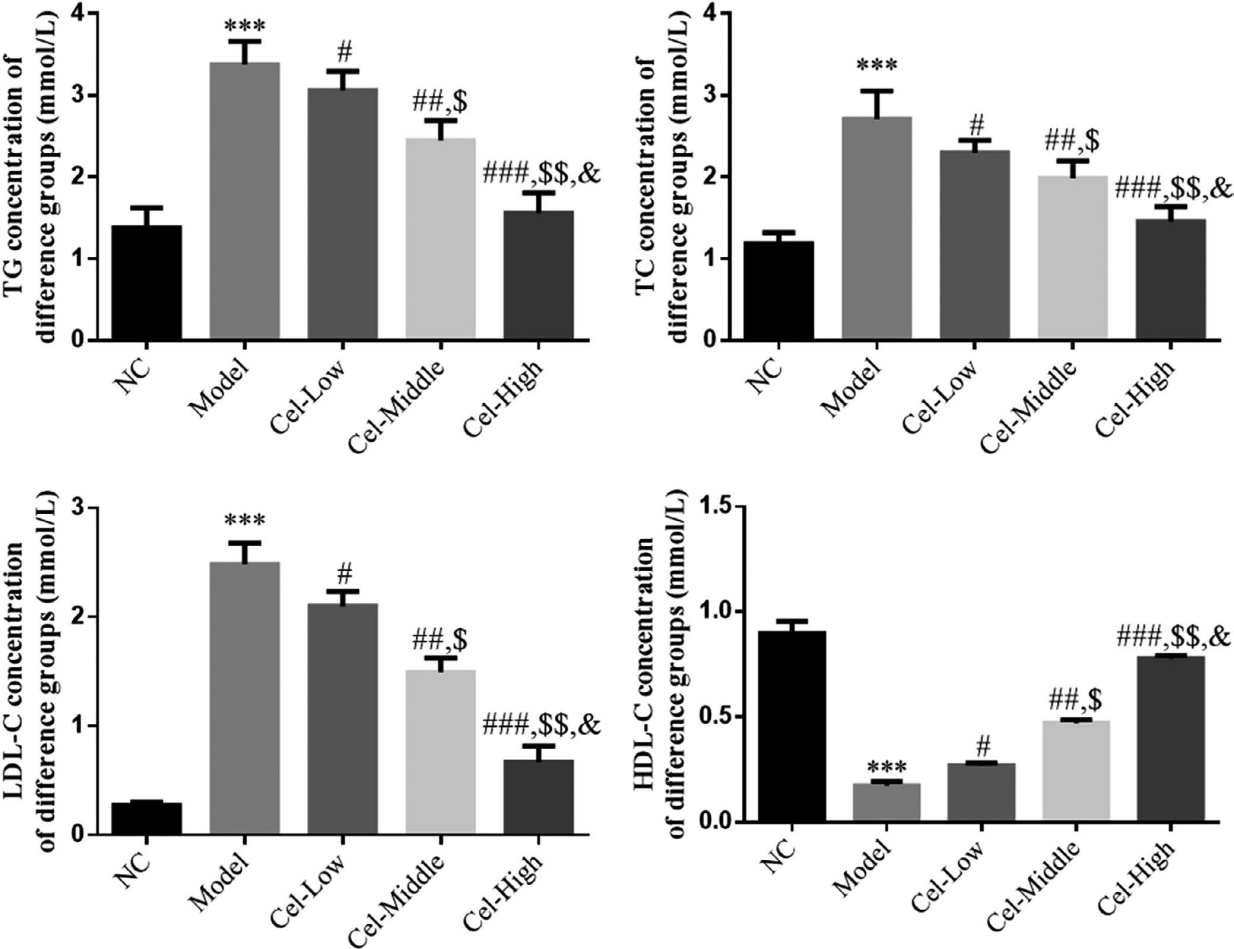
Comparison of lipid metabolism indicators in mice. NC: the mice were treated with normal; Model: T2DM mice with NAFLD mice; Cel‐Low: Model mice were treated with 10 mg/kg Cel; Cel‐Middle: Model mice were treated with 50 mg/kg Cel; Cel‐High: Model mice were treated with 100 mg/kg Cel. ***: *p *< .001, compared with NC group; #: *p *< .05, ##: *p *< .01, ###: *p *< .001, compared with Model group; $: *p *< .05, $$: *p *< .01, compared with Cel‐Low group; &: *p *< .05, compared with Cel‐Middle

Serum TG, TC, and LDL‐C levels of the Model group were significantly higher than those of the NC group, and these levels of the Cel intervention groups were significantly lower than those of the Model group (*p* < .05, Figure 2); the serum HDL‐C content of the Model group was significantly lower than that of the NC group and the level of the Cel intervention groups was significantly higher than that of the Model group (*p* < .05, Figure 2). A significant dose‐effect relationship in TG, TC, LDL‐C, and HDL‐C was observed among the three Cel groups (*p* < .05, Figure 2).

### 4.4 Comparison of liver function indexes and oxidative stress indexes

**FIGURE 3:**
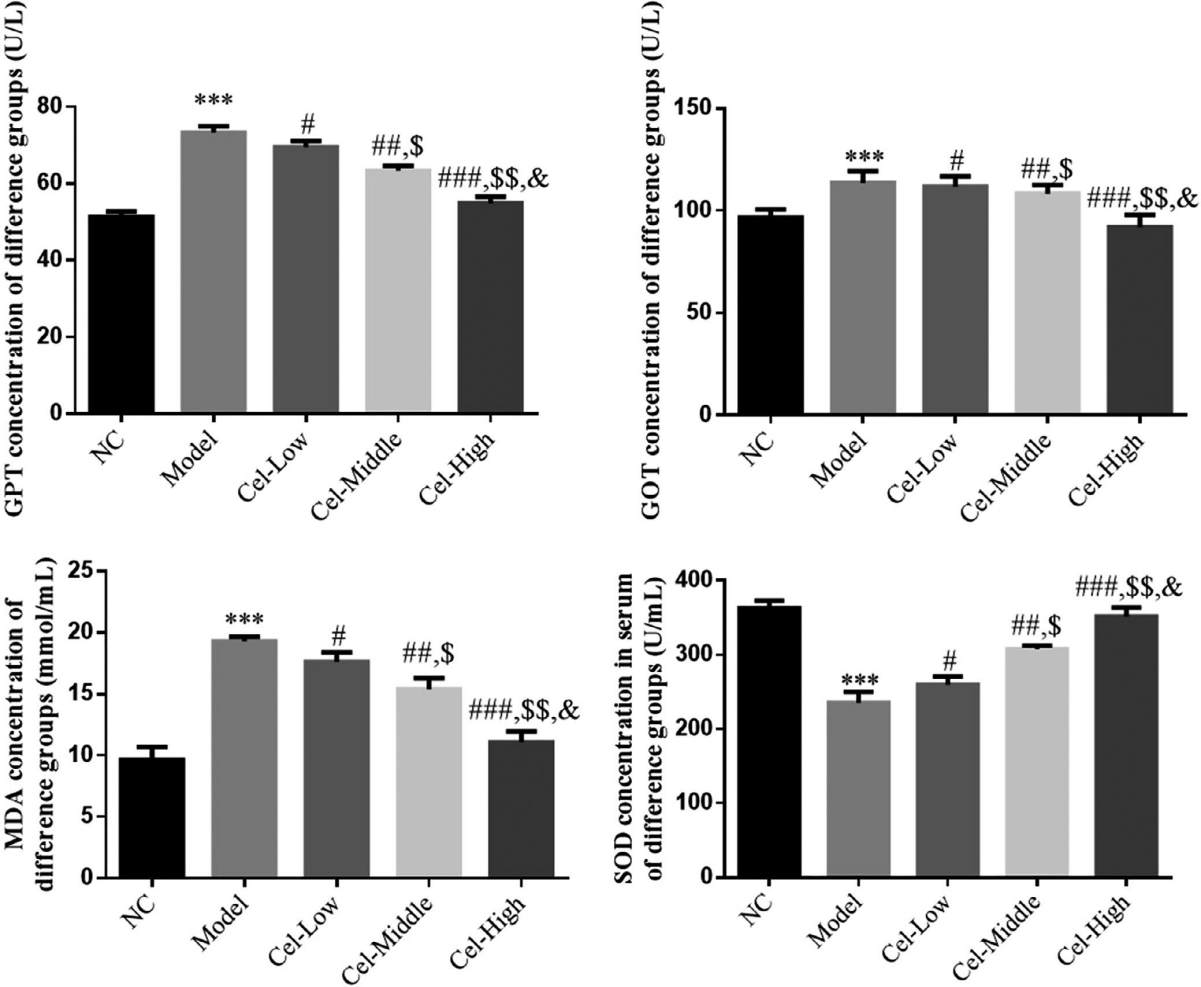
Comparison of liver function and oxidative stress index in mice. NC: the mice were treated with normal; Model: T2DM mice with NAFLD mice; Cel‐Low: Model mice were treated with 10 mg/kg Cel; Cel‐Middle: Model mice were treated with 50 mg/kg Cel; Cel‐High: Model mice were treated with 100 mg/kg Cel. ***: *p *< .001, compared with NC group; #: *p *< .05, ##: *p *< .01, ###: *p *< .001, compared with Model group; $: *p *< .05, $$: *p *< .01, compared with Cel‐Low group; &: *p *< .05, compared with Cel‐Middle

Serum GOT, GPT, and MDA levels of the Model group were significantly higher than those of the NC group (*p* < .05, Figure 3), and these levels of the Cel intervention groups were significantly lower than those of the Model group (*p* < .05, Figure 3); the serum SOD content of the Model group was significantly lower than that of the NC group, and the serum SOD content of the Cel intervention groups was significantly higher than that of the Model group (*p* < .05, Figure 3). There was a significant dose‐effect relationship among the three Cel groups in the concentrations of GOT, GPT, MDA, and SOD (*p* < .05, Figure 3).

### 4.5 Comparison of hepatocyte apoptosis

**FIGURE 4:**
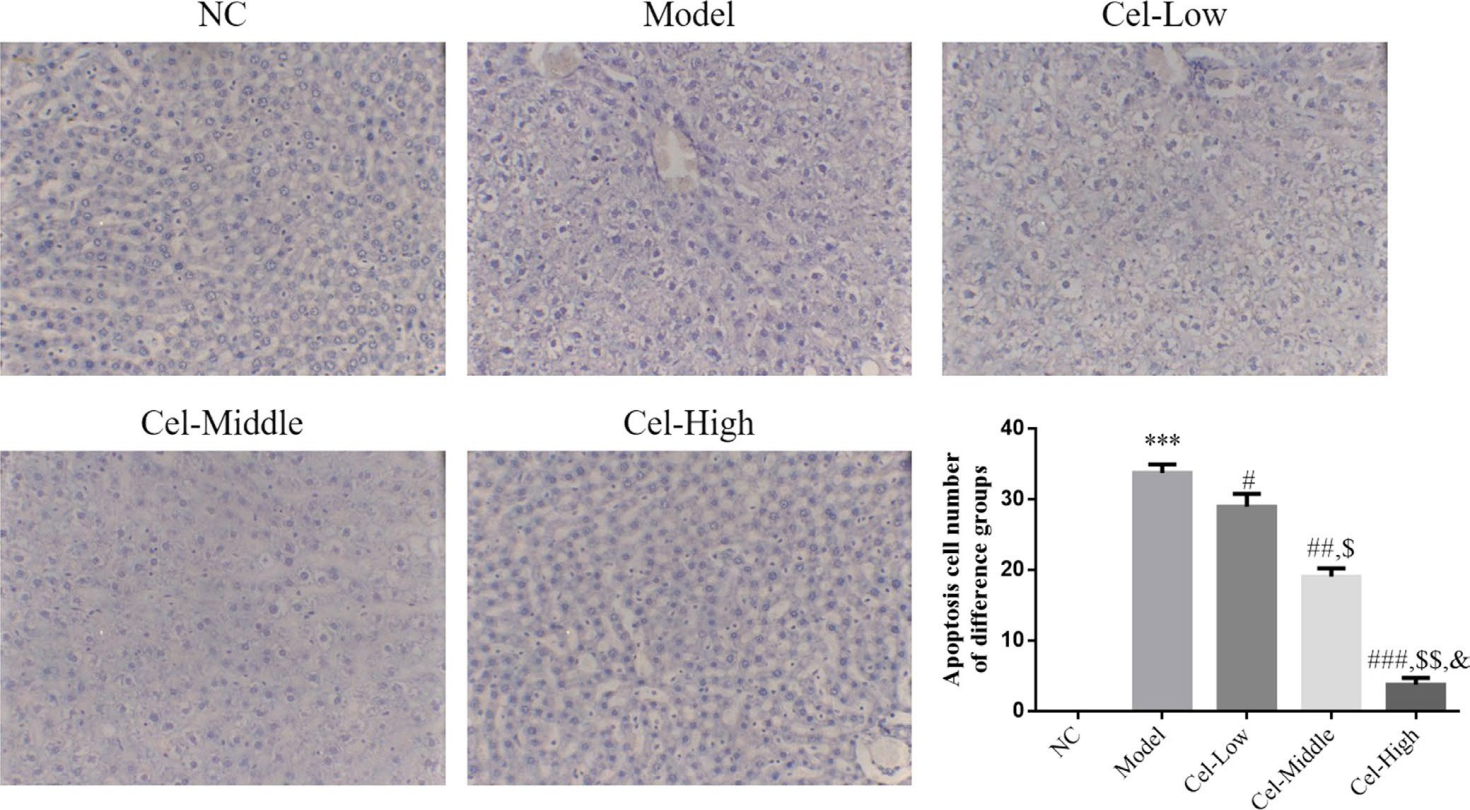
Apoptosis cell number of difference groups in liver by TUNEL assay (200×). NC: the mice were treated with normal; Model: T2DM mice with NAFLD mice; Cel‐Low: Model mice were treated with 10 mg/kg Cel; Cel‐Middle: Model mice were treated with 50 mg/kg Cel; Cel‐High: Model mice were treated with 100 mg/kg Cel. ***: *p *< .001, compared with NC group; #: *p *< .05, ##: *p *< .01, ###: *p *< .001, compared with Model group; $: *p *< .05, $$: *p *< .01, compared with Cel‐Low group; &: *p *< .05, compared with Cel‐Middle

The TUNEL staining results showed that the number of apoptotic cells in the Model group was significantly increased compared with that in the NC group (*p* < .001, Figure 4); and after treated with Cel, the number of apoptotic cells in the Cel intervention groups was significantly decreased compared with that of the Model group, (*p* < .05, Figure 4), with a significant dose‐effect relationship noted among the three Cel intervention groups (*p* < .05, Figure 4).

### 4.6 Nrf‐2 and HO‐1 proteins expressions by IHC

**FIGURE 5:**
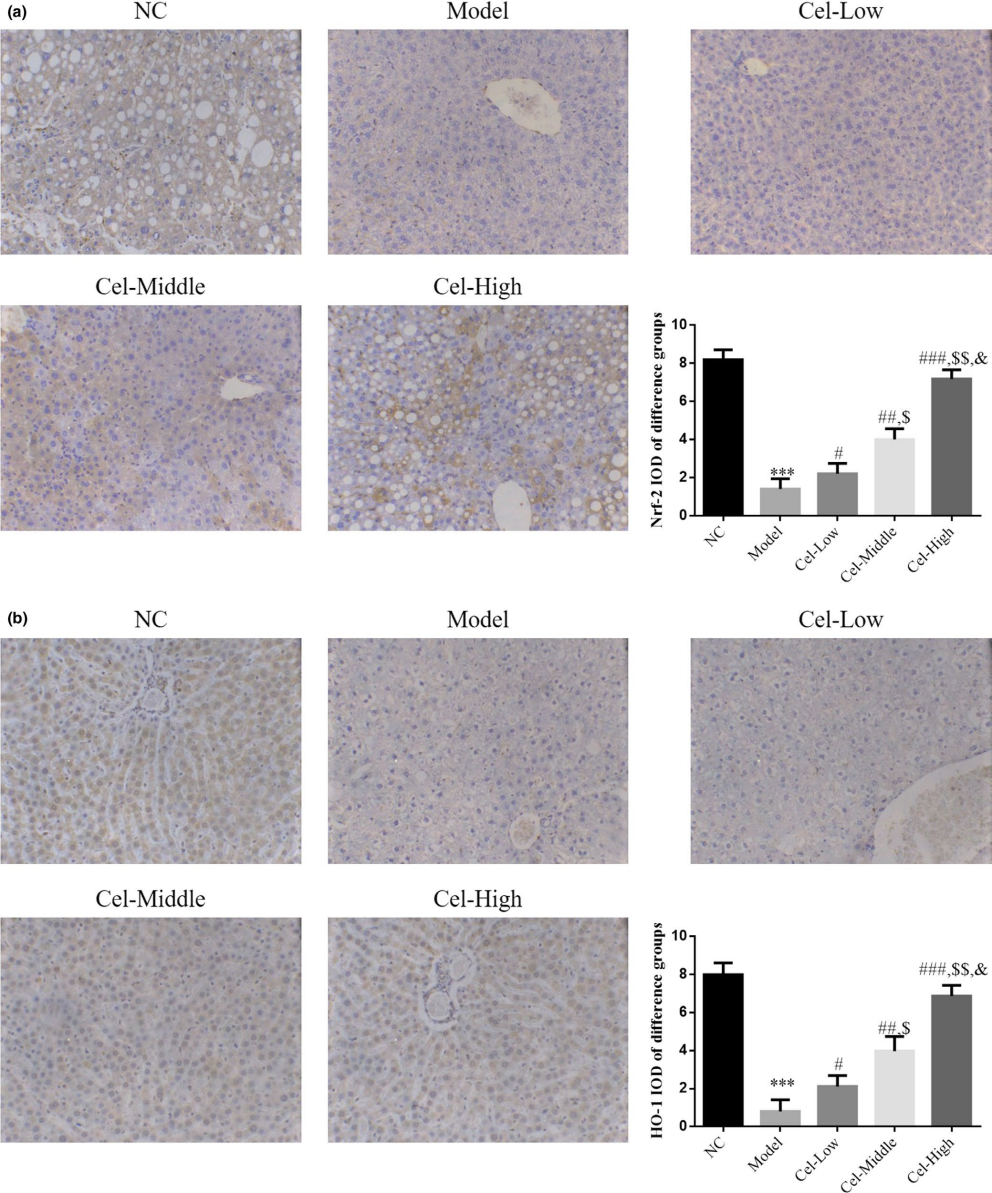
Nrf‐2 and HO‐1 protein expression by IHC assay (200×). NC: the mice were treated with normal; Model: T2DM mice with NAFLD mice; Cel‐Low: Model mice were treated with 10 mg/kg Cel; Cel‐Middle: Model mice were treated with 50 mg/kg Cel; Cel‐High: Model mice were treated with 100 mg/kg Cel. (a) Nrf‐2 protein expression in liver tissues by IHC assay (200×). ***: *p *< .001, compared with NC group; #: *p *< .05, ##: *p *< .01, ###: *p *< .001, compared with Model group; $: *p *< .05, $$: *p *< .01, compared with Cel‐Low group; &: *p *< .05, compared with Cel‐Middle. (b) HO‐1 protein expression in liver tissues by IHC assay (200×). ***: *p *< .001, compared with NC group; #: *p *< .05, ##: *p *< .01, ###: *p *< .001, compared with Model group; $: *p *< .05, $$: *p *< .01, compared with Cel‐Low group; &: *p *< .05, compared with Cel‐Middle

The IHC staining results demonstrated that the expression levels of Nrf‐2 and HO‐1 proteins were significantly reduced in the Model group compared with those in the NC group, respectively (*p* < .001 for all comparisons, Figure 5); after treated with different concentrations of Cel, the expressions of Nrf‐2 and HO‐1 proteins in the Cel intervention groups were significantly increased compared with those of the Model group(*p* < .05 for all, Figure 5), and a significant dose‐effect relationship was observed among the Cel intervention groups (*p* < .05, Figure 5).

### 4.7 Gene and protein expressions of Nrf‐2 and HO‐1

**FIGURE 6:**
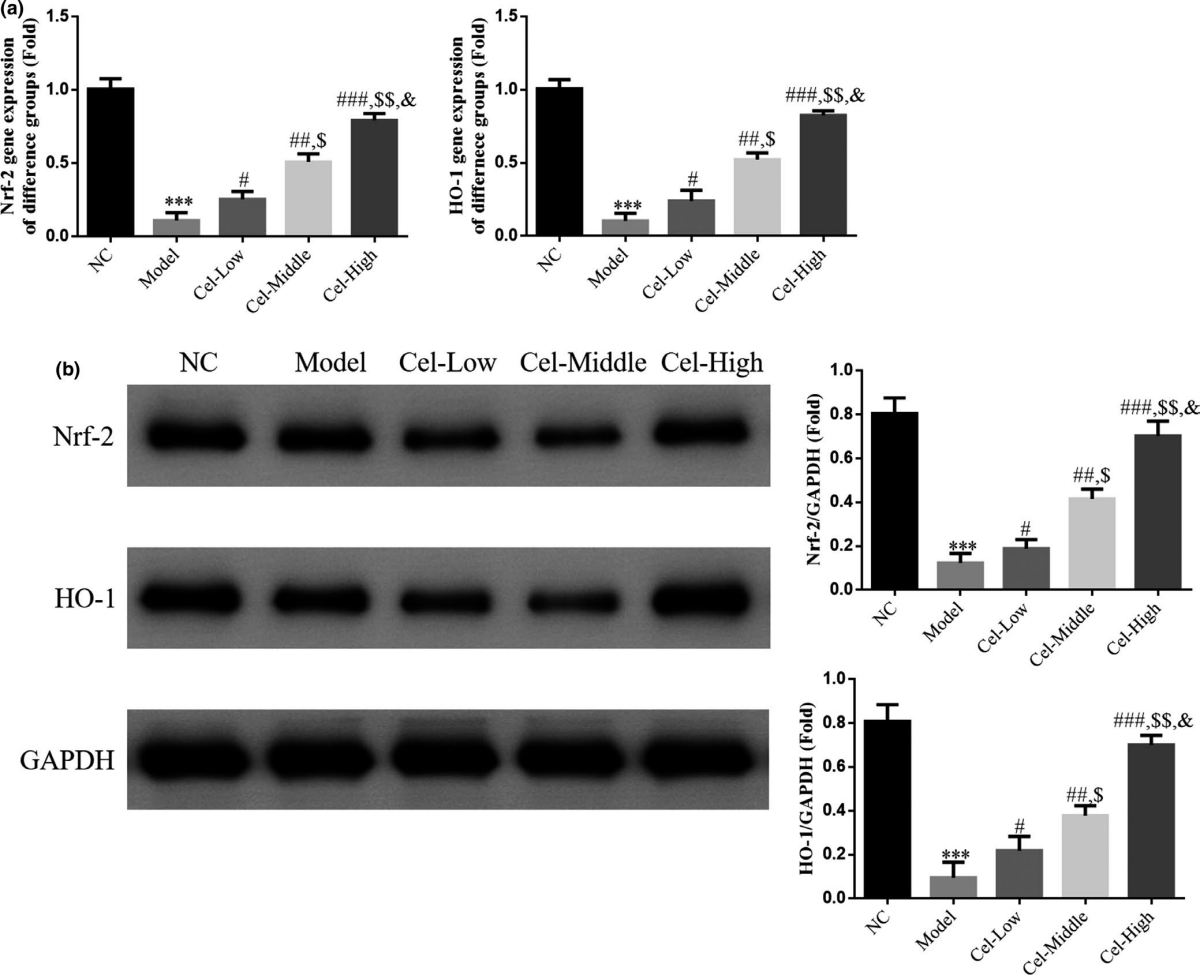
Nrf‐2 and HO‐1 mRNA and protein expression in difference groups. NC: the mice were treated with normal; Model: T2DM mice with NAFLD mice; Cel‐Low: Model mice were treated with 10 mg/kg Cel; Cel‐Middle: Model mice were treated with 50 mg/kg Cel; Cel‐High: Model mice were treated with 100 mg/kg Cel. (a) Nrf‐2 and HO‐1 mRNA expression of difference groups by RT‐PCR assay. ***: *p *< .001, compared with NC group; #: *p *< .05, ##: *p *< .01, ###: *p *< .001, compared with Model group; $: *p *< .05, $$: *p *< .01, compared with Cel‐Low group; &: *p *< .05, compared with Cel‐Middle. (b) Nrf‐2 and HO‐1 protein expression by WB assay. ***: *p *< .001, compared with NC group; #: *p *< .05, ##: *p *< .01, ###: *p *< .001, compared with Model group; $: *p *< .05, $$: *p *< .01, compared with Cel‐Low group; &: *p *< .05, compared with Cel‐Middle

RT‐PCR and WB analysis showed that the expression levels of Nrf‐2 and HO‐1 genes and proteins were significantly reduced in the model group compared with those of the NC group (*p* < .001 for all, Figure 6); and after treated with different concentrations of Cel, the expressions of Nrf‐2 and HO‐1 genes and proteins in the Cel intervention groups were significantly increased compared with those of the Model group (*p* < .05 for all, Figure 6) with a significant dose‐effect relationship noted among the Cel intervention groups (*p* < .05, Figure 6).

## 5 DISCUSSION

T2DM is highly correlated with NAFLD (Hazlehurst et al., 2016; Morrison et al., 2019). T2DM can further induce NAFLD and vice versa (Bril & Cusi, 2016). This can be explained by the fact that T2DM causes insulin resistance and promote liver lipid deposition, and excessive lipid deposition exacerbates lipid oxidation and therefore enhances insulin resistance. The interaction between T2DM and NAFLD results in aggravation of the disease, which threatens life and health (Pierantonelli & Svegliati‐Baroni, 2019).

The pathogenesis of T2DM complicated with NAFLD has not been fully addressed. Studies have shown that glucotoxicity, lip toxicity, and resultant chronic inflammation are involved in the development of these diseases (Sokolovska et al., 2015). The liver is the main place for fat metabolism, where excessive fat metabolism produces massive amount of oxygen free radicals that induce lipid peroxidation, resulting in oxidative stress and inflammatory factor infiltration. Continued inflammation may further induce insulin resistance, and as a result, T2DM with NAFLD progresses (Buzzetti et al., 2016). Therefore, anti‐inflammation and antioxidation are the key to treat T2DM with NAFLD and improve hepatocyte apoptosis.

Studies have shown that Cel can reduce focal cerebral ischemia‐reperfusion injury in rats, and the underlying mechanism may involve the inhibition of NF‐κB activation, the expression of TNF‐α and IL‐1β, and the subsequent reduction of inflammation (Zhang et al., 2018). Yu et al. (2017) demonstrated that Cel can inhibit the activation of NLRP3 inflammasomes and the expression of downstream Caspase‐1 and IL‐1β in vivo and in vitro, suggesting that Cel is effective in the treatment of NLRP3 inflammasome‐dependent inflammatory diseases. Other studies have found that Cel enhances intracellular ATP content, mitochondrial membrane potential and fatty acid oxidation, thereby improving the biological function of mitochondria. In addition, Cel also increases intracellular ATP content, maintains mitochondrial membrane potential, enhances citrate synthase activity, and reduces mitochondrial superoxide, thus improving the function of mitochondria (Bakar & Tan, 2017; Xiaowen et al., 2018). In the present study, pathological injury in liver tissue and hepatocyte apoptosis due to increased lipid levels and activation of oxidative stress were observed in T2DM with NAFLD mice. After the treatment with Cel, the pathological injury of liver tissue and hepatocyte apoptosis were significantly improved. Furthermore, the oxidative stress‐related pathways were investigated to further explore the mechanisms involved.

Nuclear transcription factor Nrf2 is a receptor for oxidative stress in cells. As an important transcription factor that regulates the antioxidative stress response, Nrf2 plays an essential role in the defense mechanism of antioxidative stress. Nrf2 binds to Keapl, an actin‐binding protein, to up‐regulate the expression of OH‐1, activate the Keapl/Nrf2/OH‐1 pathway, increase the activities of antioxidant enzymes such as superoxide dismutase and glutathione peroxidase, remove harmful substances including oxygen free radicals, and maintain the redox balance of the body to protect cells (Wu et al., 2020). At rest, the formed Kepl‐Nrf2 dimer locates in the cytoplasm. When stimulated by reactive oxygen species or external oxidative stress signals, Nrf2 is phosphorylated and translocated into the nucleus, where it exerts the antioxidant activity via activating the expression of downstream OH‐1 and SOD (Li et al., 2019). In the present study, the expression levels of Nrf‐2 and HO‐1 in the liver tissue of T2DM with NAFLD mice were significantly reduced, which may be the key factor leading to the injury and apoptosis of hepatocytes. After Cel intervention, the Nrf‐2/HO‐1 signaling pathway was activated to protect liver tissue and cells.

In conclusion, Cel improves lipid metabolism and inhibits oxidative stress to protect liver tissue and cells in T2DM with NAFLD mice. The potential mechanism involves the upregulation of Nrf‐2 and OH‐1, elimination of free radicals, and improvement in lipid peroxidation.
